# Integrated Findings on Geriatric Healthcare Utilization Among Older Jewish and Arab Adults in Israel

**DOI:** 10.1093/geroni/igaf122.2589

**Published:** 2025-12-31

**Authors:** Efrat Gil, Rachel Kizony, Roy Tzemah-Shahar, Maayan Agmon

**Affiliations:** Israel Ministry of Health, Jerusalem, Yerushalayim, Israel; University of Haifa, Haifa, HaZafon, Israel; University of Haifa, Haifa, HaZafon, Israel; University of Haifa, Haifa, HaZafon, Israel

## Abstract

Israel’s aging population presents healthcare challenges, with disparities between Jewish and Arab older adults. While 88% of those aged 65+ in Israel are Jewish and 8% Arab, the latter group has lower life expectancy due to higher morbidity, reduced preventive care, and limited healthcare use. Arabs rely more on primary care and hospitalizations, often seeking medical attention only when health worsens. This study explored geriatric healthcare utilization patterns among community dwelling Jewish and Arab older adults, identifying barriers and disparities. The research included three phases: (1) a focus group with six primary care professionals to identify barriers to geriatric service use, (2) quantitative and qualitative data collection from 401 older adults (mean age 74.9±6.4 years) assessing service utilization, and (3) a validation focus group with eight healthcare professionals. Findings reveal low awareness of geriatric services across both populations, with many respondents unsure why people seek or avoid care. Reasons for seeking services included eligibility for financial support, severe health deterioration, and referrals from healthcare providers. Barriers included lack of awareness, stigma, and misconceptions about geriatric services. Notably, Jewish participants were more proactive in seeking geriatric consultations, while Arab participants relied primarily on referrals from family doctors. Additionally, healthcare professionals and participants highlighted long waiting times, a shortage of geriatric specialists, and confusion about when to seek geriatric versus primary care, further complicating service utilization. Policy recommendations emphasize expanding multidisciplinary geriatric care, increasing accessibility and awareness, addressing healthcare disparities, and implementing proactive geriatric assessments ensuring early intervention and improve health outcomes.

